# Biological Insights into Chemotherapy Resistance in Ovarian Cancer

**DOI:** 10.3390/ijms20092131

**Published:** 2019-04-30

**Authors:** Michelle A. Glasgow, Peter Argenta, Juan E. Abrahante, Mihir Shetty, Shobhana Talukdar, Paula A. Croonquist, Mahmoud A. Khalifa, Timothy K. Starr

**Affiliations:** 1 Department of Obstetrics, Gynecology & Women’s Health, University of Minnesota, Minneapolis, MN 55455, USA; michelle.a.glasgow@gmail.com (M.A.G.); argenta@umn.edu (P.A.); shett036@umn.edu (M.S.); taluk009@umn.edu (S.T.); 2 Masonic Cancer Center, University of Minnesota, Minneapolis, MN 55455, USA; abrah0023@umn.edu; 3 Department of Biology, Anoka Ramsey Community College, Coon Rapids, MN 55455, USA; Paula.Croonquist@anokaramsey.edu; 4 Department of Laboratory Medicine and Pathology, University of Minnesota, Minneapolis, MN 55455, USA; mkhalifa@umn.edu

**Keywords:** ovarian cancer, chemotherapy resistance, gene expression

## Abstract

The majority of patients with high-grade serous ovarian cancer (HGSOC) initially respond to chemotherapy; however, most will develop chemotherapy resistance. Gene signatures may change with the development of chemotherapy resistance in this population, which is important as it may lead to tailored therapies. The objective of this study was to compare tumor gene expression profiles in patients before and after treatment with neoadjuvant chemotherapy (NACT). Tumor samples were collected from six patients diagnosed with HGSOC before and after administration of NACT. RNA extraction and whole transcriptome sequencing was performed. Differential gene expression, hierarchical clustering, gene set enrichment analysis, and pathway analysis were examined in all of the samples. Tumor samples clustered based on exposure to chemotherapy as opposed to patient source. Pre-NACT samples were enriched for multiple pathways involving cell cycle growth. Post-NACT samples were enriched for drug transport and peroxisome pathways. Molecular subtypes based on the pre-NACT sample (differentiated, mesenchymal, proliferative and immunoreactive) changed in four patients after administration of NACT. Multiple changes in tumor gene expression profiles after exposure to NACT were identified from this pilot study and warrant further attention as they may indicate early changes in the development of chemotherapy resistance.

## 1. Introduction

About 85% of patients with high-grade serous ovarian cancer (HGSOC) will achieve a clinical remission with a combination of surgery and platinum-based chemotherapy despite presenting with advanced stage disease [1]. Though many will respond to additional rounds of treatment and may experience prolonged remission, disease-free intervals generally become progressively shorter, culminating in platinum and ultimately chemotherapy resistance, suggesting a fundamental and progressive change in the biology of the tumor. 

The development of chemotherapy resistance is hypothesized to occur through multiple routes, most notably selection of resistant clones and upregulation of tumor-protective pathways. To elucidate the mechanisms underlying platinum resistance several groups have generated chemoresistant versions of established ovarian cancer cell lines and compared gene expression between the chemoresistant line and its chemosensitive parental line [2,3,4,5]. These studies provide insight into changes that occur as cells adapt to high levels of chemotherapy, but the genes identified in these studies are inconsistent between studies and the mechanisms of in vitro resistance could very likely be different from those arising in human tumors [6]. 

Similarly, comparisons of gene expression between patients who achieved clinical remission versus those who were refractory identified expression signatures of chemoresistance, but patient heterogeneity was a noted confounding factor in these comparisons [5,7]. To eliminate interpatient heterogeneity, Patch et al. compared matched samples taken from patients at initial debulking and at recurrence [8], suggesting that gene profiles in the latter may represent a chemoresistant gene signature.

A large systematic review of 42 studies attempting to define molecular signatures that predict resistance to chemotherapy in ovarian cancer found that gene signatures were not consistent between studies and concluded that there are no gene signatures currently appropriate for clinical use [9]. With larger datasets becoming available, it may be possible to improve upon previous studies. For example, Yin et al. used TCGA data to devise a 131-gene signature correlating with platinum resistance [10]. 

Another approach for predicting the response to chemotherapy is to perform in vitro growth assays using fresh tumor samples exposed to chemotherapy [11]. The sensitivity and specificity of these in vitro assays vary, but they do show promise as a possible means to predict patient response, although they have not yet had widespread use in a clinical setting [12,13,14].

A goal of precision medicine is to classify patients based on the genomic and transcriptomic characteristics of their tumor and use these classifications to guide treatment decisions. This approach has been successful in breast cancer, where gene expression signatures can be used for prognosis and to predict chemotherapy response [15,16]. There are now gene-expression-based lab diagnostics that are routinely used to stratify breast cancer patients into molecular subtypes and guide treatment options [17,18]. In ovarian cancer, several large-scale datasets have been used to stratify ovarian cancer patients into molecular subtypes based on gene expression [19,20,21,22]; this stratification has prognostic and therapeutic relevance [23,24], but has not yet been prospectively validated for clinical use [25]. 

The aim of this study was to compare gene expression profiles for individuals before and early into the course of treatment in hopes of identifying early changes in expression that may herald either up-front chemoresistance or provide insights into the sequence of events involved in the development of resistance. Patients undergoing neoadjuvant chemotherapy (NACT), who typically require a biopsy for diagnosis followed by an interval cytoreduction, were felt to be ideal to address these questions.

## 2. Results

### 2.1. Patient Characteristics and RNA Sequencing Metrics

**Figure 1:**
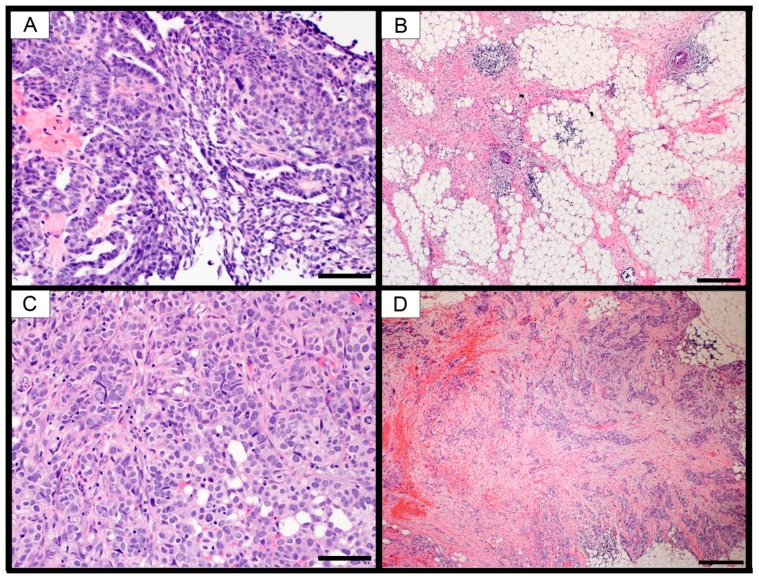
Photomicrographs of selected cases showing high-grade serous carcinoma. (**A**) Patient #17, high-power magnification of pre-NACT omental biopsy showing solid clusters of malignant cells with high purity and no intervening stroma. (**B**) Low-power magnification of post-NACT omental biopsy from the same patient showing CRS of 3. (**C**) Patient #1, high-power magnification of pre-NACT omental biopsy showing sheets of malignant cells with high purity and no intervening stroma. (**D**) Low-power magnification of post-NACT omental biopsy from the same patient showing CRS of 2. Scale bars: A and C = 100 µm, B and D = 500 µm.

Six patients diagnosed with HGSOC were enrolled in this study. We collected pre-NACT samples from these patients either via CT-guided biopsy or intraoperatively. We collected post-NACT samples at the time of interval debulking surgery (IDS). The baseline characteristics, chemotherapy regimens, disease status, platinum response classification, sample site, chemotherapy response score (CRS), and change in tumor purity ESTIMATEScore comparing pre- to post-NACT samples for all patients are shown in Table 1. All patients received at least three cycles of NACT. We performed RNA sequencing on all of the matched pre- and post-NACT samples. Of note, four patients were platinum-resistant and two were platinum-sensitive based on clinical evidence of disease recurrence within six months of the final administration of chemotherapy. The two platinum-sensitive patients also had CRS scores of 3, indicating early response to platinum therapy. The CRS score represents a systematic histopathologic assessment of response to NACT [26,27]. Tumor purity and CRS scores were evaluated by an expert clinical pathologist specializing in ovarian cancer (Figure 1 and Table 1). All tumors had high purity of tumor cells compared to stroma, based on the evaluation of hematoxylin and eosin stained sections by a clinical pathologist (Figure 1). Yoshihara et al. developed a bioinformatic algorithm for estimating tumor purity based on gene expression data [28]. This algorithm produces an “ESTIMATEScore” based on expression of a subset of genes. When applied to 248 ovarian samples from TCGA patients, ESTIMATEScores ranged from −3647 to +3205, with a lower score indicative of higher purity. We performed the ESTIMATE algorithm and the ESTIMATEScores ranged from −566 to 4361 (Figure S1). The differences between the pre- and post-NACT ESTIMATEScores varied by patient (Table 1 and Figure S1).

We obtained an average of 23.7 million reads per sample, with greater than 95% of reads mapping to the genome and an average or 20,459 genes detected per sample. We limited our analysis to well-annotated genes with average expression levels above 5 FPKM.

### 2.2. Chemotherapy Effects on Gene Expression Are Stronger than the Effects of Inter-Patient Heterogeneity

**Figure 2:**
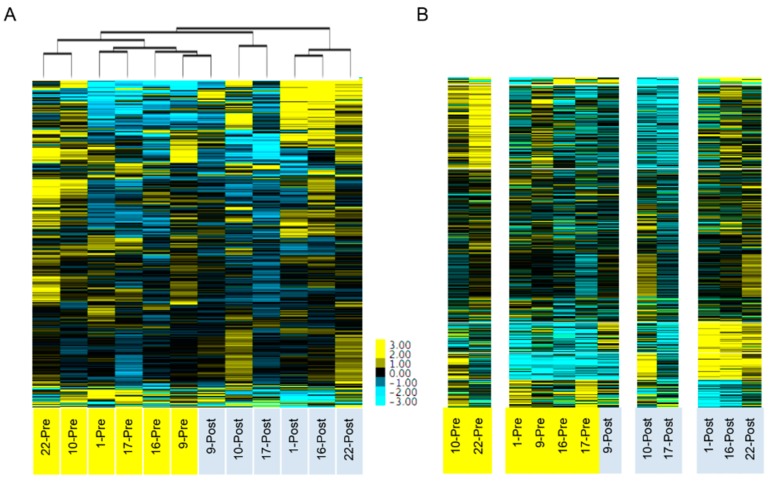
Unsupervised hierarchical clustering and K-means clustering. Clustering of samples using the top 366 highly variable genes (average deviation > 50). (**A**) Unsupervised hierarchical clustering with dendrogram. (**B**) K-means clustering (k = 4). Samples are labeled by patient ID numbers and Pre or Post, depicting pre-NACT and post-NACT samples.

**Figure 3:**
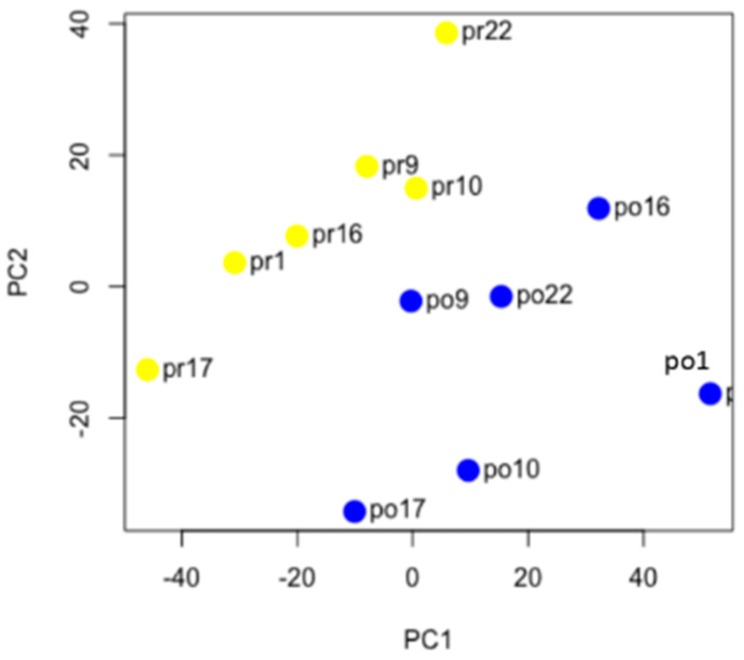
Principal component analysis of pre-NACT and post-NACT samples. Principal component analysis was performed using the top 6748 expressed genes in the 12 samples. Yellow dots are pre-NACT samples and blue dots are post-NACT samples. Patient ID numbers are listed.

The genomic landscape of ovarian cancer is characterized by extensive copy number variation (CNV), which affects gene expression [29,30]. There are recurrent chromosomal regions of CNV in ovarian cancer that are common to many patients, but patients may also have “private” CNVs that are specific to that patient. We predicted that the pre-NACT and post-NACT samples for each patient would have similar gene expression patterns. However, chemotherapy can also affect gene expression patterns. To determine which of these has a stronger effect on gene expression (chemotherapy or interpatient heterogeneity), we performed unsupervised hierarchical clustering and k-means clustering. Using a set of 366 genes that had the most highly variable expression between the samples (average deviation > 50), we found that, with one exception (patient 9), samples clustered based on their chemotherapy status and not based on their patient source (Figure 2). Principal component analysis using 6748 consistently expressed genes (minimum FPKM > 5) also resulted in clustering by chemotherapy status and not by patient source (Figure 3). Together, these results suggest that the effects of chemotherapy on gene expression are stronger than the differences in gene expression between patients and further suggest a commonality of response to the stress of chemotherapy. 

### 2.3. Chemotherapy Changes the Molecular Subtype of the Patient

Several groups have defined HGSOC molecular subtypes using gene expression datasets from samples taken from chemo-naïve patients during their initial debulking surgery [19,20,24]. In TCGA analysis, based on the set of genes upregulated within each of these clusters, the molecular subtypes have been given the names Differentiated, Proliferative, Mesenchymal, and Immunoreactive. These clustering patterns are generated using a subset of genes that are highly expressed and highly variable and applying various clustering algorithms. The initial TCGA clusters were generated using a clustering method referred to as non-negative matrix factorization (NMF) on a set of ~800 genes [20]. 

**Figure 4:**
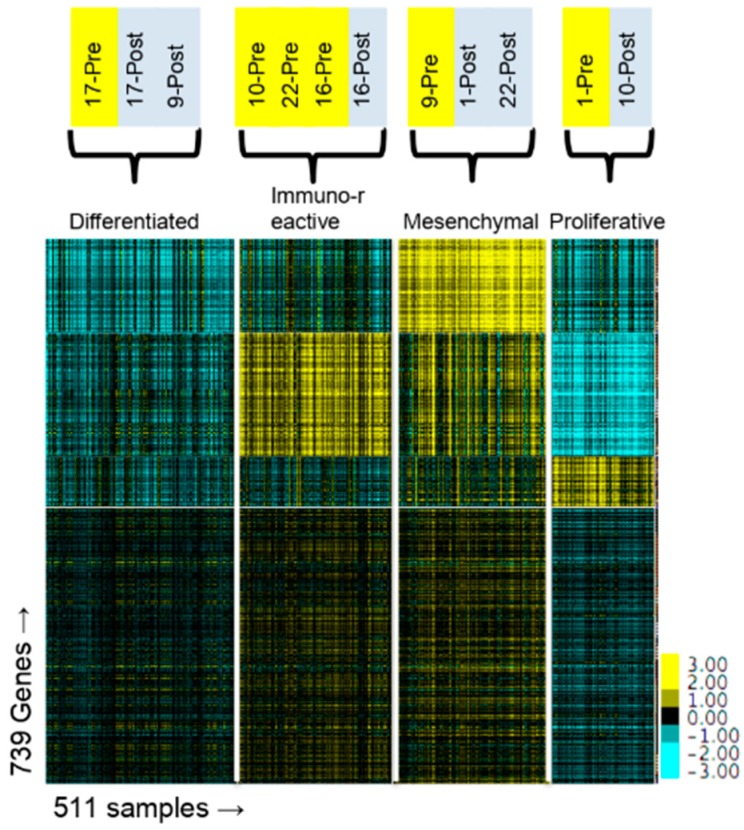
NMF clustering of TCGA, pre-NACT and post-NACT samples. Clustering of NMF target matrix containing combined RNASeq gene expression data from TCGA (*n* = 499), pre-NACT (*n* = 6) and post-NACT (*n* = 6) samples (k = 4). TCGA molecular subtype name is indicated at the top of the heat map. Pre- and Post-NACT sample molecular group assignment is indicated at the top of the heat map.

Having documented the effect of chemotherapy on gene expression, we predicted that chemotherapy would potentially alter the molecular subtype designation of our patients. To determine the molecular subtypes of our 12 samples, we combined RNAseq data from TCGA patients with our 12 samples and performed NMF using the same set of ~800 genes. Because the original clustering of TCGA patients was done using microarray data, we first checked to see if clustering with RNASeq data produced similar clusters. Using RNASeq data, 84% of patients were classified in the same molecular subtype as originally reported (Table S1). In our combined dataset, of the six pre-NACT samples, three were classified as immunoreactive and one each as proliferative, mesenchymal and differentiated (Table 2 and Figure 4). As we predicted, the molecular subtype for four of the six patients changed after administration of chemotherapy. Two of three patients that were initially classified as immunoreactive switched, one each to proliferative and mesenchymal (patients 10 & 22). Two additional patients switched from proliferative and mesenchymal subtypes (patients 1 & 9) (Table 2). These findings indicate that chemotherapy causes significant changes to gene expression patterns resulting in changes in the molecular subtyping of the tumor.

### 2.4. Cell Cycle Pathways Are Enriched in Pre-NACT Samples

To identify important biological differences between pre- and post-NACT samples, we identified signaling pathways and biological states enriched in the pre-NACT samples compared to the post-NACT samples. For our first approach, we performed gene set enrichment analysis (GSEA) using a collection of 50 “hallmark” gene sets representing well-defined biological states or processes [31,32]. Hallmark gene sets relating to cell cycle and cell growth were significantly enriched in the pre-NACT samples compared to the post-NACT samples. Of the 10 significantly (*p* < 0.01) enriched hallmark gene sets, five were cell cycle pathways (G2M-Checkpoint, E2F-targets, Mitotic-Spindle, MYC V1, and MYC V2-Targets). Another three enriched hallmark gene sets were related to growth (Glycolysis, MTORC1-Signaling, and PI3K-AKT-MTOR-Signaling), while the remaining two enriched gene sets were DNA-Repair and the Unfolded-Protein-Response (Table 3).

To confirm these results, we used a second, complementary approach to identify pathways enriched in the pre-NACT samples. First, we identified differentially expressed genes using the software package EdgeR, selecting genes that were at least 1.5-fold higher in pre-NACT samples (Poisson model FDR < 0.0001) [33]. This analysis identified 117 genes that were significantly higher in pre-NACT samples compared to post-NACT samples (Table S2). We performed over-representation analysis based on this set of genes using ConsensuPathDB [34]. ConsensusPathDB tests for over-representation of the gene set in over 4000 signaling pathways extracted from 12 annotated pathway databases including KEGG, Pathway Interaction Database, Reactome, and Wikipathways. We identified 86 pathways significantly enriched with our gene set (Table S3, Hypergeometric test, *p* < 0.001). Similar to the GSEA results, ~60% of significantly over-represented pathways related to the cell cycle. The overexpressed genes contributing to this enrichment included several cyclins (*CCNB1*, *CCNB2*, *CCNA1*), cell division control genes (*CDC20*, *CDC25A*, *CDC45*), a cyclin dependent kinase (*CDK1*), regulators of mitosis (*AURKB*, *PLK1*, *CENPF*, *BUB1*) and other key regulators of the cell cycle (*CHEK1*, *E2F2*). Using the same set of 117 genes, we also tested for enriched terms from Gene Ontology (GO) database. This analysis resulted in identification of 37 GO terms, again with ~60% being cell-cycle-related (Table S4).

### 2.5. Drug Transport and Peroxisome Pathways Are Enriched Post-NACT Samples

**Figure 5:**
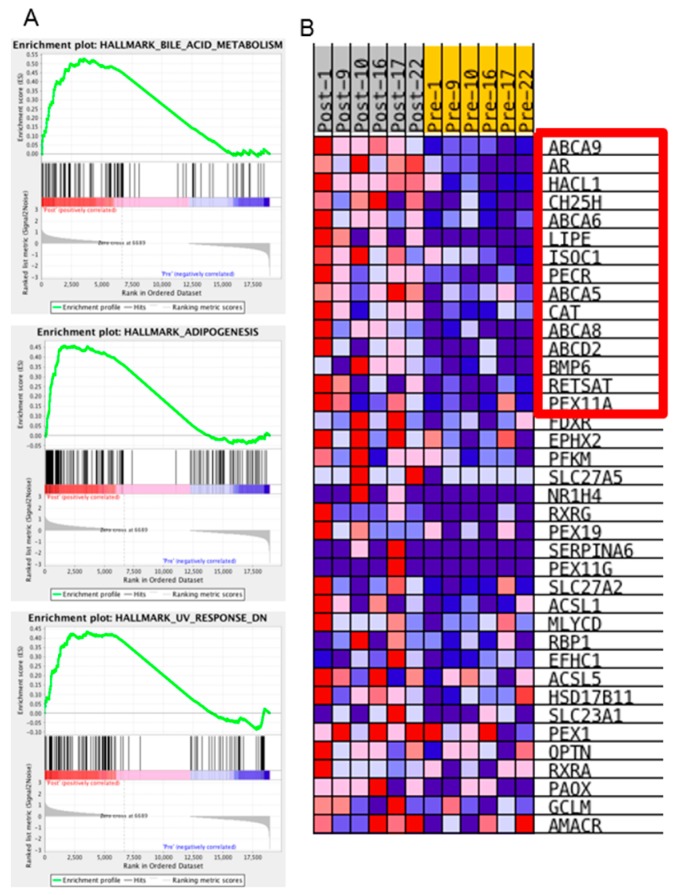
GSEA Post-NACT enrichment plot for genes related to bile acid metabolism. Gene sets enriched for genes upregulated in post-NACT samples based on GSEA analysis. (**A**) Post-NACT enrichment plots for the three significantly enriched Hallmark gene sets, Bile Acid Metabolism, Adipogenesis and UV response down (*p* < 0.01 based on comparison to ESnull dataset, see methods). (**B**) Heatmap of the 38 genes contributing to bile acid metabolism GSEA enrichment plot. A red box is drawn around the top 15 upregulated genes.

Post-NACT samples were also subjected to GSEA analyses, but interestingly, only three hallmark gene sets were significantly enriched in post-NACT samples (Figure 5A and Table 4). The most significantly enriched hallmark gene set was Bile Acid Metabolism, which is a synthesis of 28 annotated pathways. In addition to bile acid metabolism genes, this hallmark gene set includes drug transporter, peroxisome, and drug response pathways. To understand the functional significance of this pathway to post-NACT samples, we analyzed the function of the top 15 genes upregulated in post-NACT samples that resulted in this pathway scoring as significant (Figure 5B and Table 4). None of the 15 genes were directly related to bile acid metabolism. Instead, one-third of the genes were drug transporters (*ABCA5*, *ABCA6*, *ABCA8*, *ABCA9*, and *ABCD2*) but none of these genes were in the MDR/TAP family of ABC drug transporters, which includes *ABCB1* (also known as *MDR1*). Previous studies have implicated upregulation of *ABCB1* as a mechanism for both platinum and taxane resistance [8,35,36]. In our dataset *ABCB1* expression was very low in all samples (Table S5). In addition to drug transporters, one-third of the upregulated genes from the Bile Acid Metabolism gene set were involved in fatty acid or cholesterol metabolism and were connected with functioning of the peroxisome (*HACL1*, *CH25H*, *LIPE*, *PECR*, and *PEX11A*) (Figure 5B and Table 4). Supporting the importance of the peroxisome, an activator of peroxisome proliferation, PPARG, is significantly upregulated in the post-NACT samples compared to pre-NACT samples (Fold Change > 1.5, FDR < 0.001, Table S6). 

We identified 266 genes upregulated in post-NACT samples using the complementary approach (Table S6). An analysis of this gene list for enriched pathways using ConsensusPathDB identified 47 enriched pathways from eight annotated pathway databases (Table S7). The most significantly enriched pathways included lipid metabolism, adipogenesis, PPAR signaling, drug metabolism and bile acid metabolism, similar to the hallmarks analysis. Analysis of GO term enrichment resulted in somewhat contradictory terms being highly enriched (Table S8). For example, the top 10 enriched GO terms included “negative regulation of cell proliferation,” “positive regulation of cell proliferation,” “regulation of lipid metabolic process,” and “negative regulation of lipid metabolic process.” 

### 2.6. Platinum Resistant Tumors Are Characterized by Lower Levels of Membrane Transporters

To understand the difference between patients that respond to platinum chemotherapy compared to those that recur within six months, we identified genes that were upregulated or downregulated in the clinically defined resistant patients compared to the sensitive patients. Due to the small sample size (two sensitive and four resistant patients), we used stringent statistical requirements for a gene to be considered up- or downregulated and we compared the set of pre-NACT samples separately from the post-NACT samples (FDR *p*-value < 0.05 after Benajamini‒Hochberg correction, see Section 4.4). In the pre-NACT samples, there were only 10 significantly downregulated genes and no upregulated genes in the resistant samples (Table S9). Of the 10 downregulated genes, three were of unknown function, two genes encode transcription factors (*FOXA1* and *ZNF648*), while one gene regulates glycogenesis (*GYG2*). The remaining four genes encode membrane proteins that regulate calcium channels, extracellular levels of ATP and biogenesis of collagen fibrils (*CACNA1E, PKD1L2, ENTPD3*, and *EPYC*). 

In the post-NACT samples there was a larger number of differentially expressed genes when comparing the sensitive to the resistant samples. Using the same stringent criteria, there were 40 downregulated genes and five upregulated genes in the resistant samples. Four of the five upregulated genes encode for nuclear proteins that regulate transcription and MAPK signaling (*ID3*, *IER2*, *GADD45B*, and *DUSP5*). The other upregulated gene, *CYR61*, is a secreted protein that interacts with the extracellular matrix to promote cell proliferation, chemotaxis, angiogenesis, and cell adhesion (Table S10). 

Of the 45 genes that are expressed at significantly lower levels in resistant patients, almost half are of unknown function. There were two downregulated transcription factors (*BARX2* and *CDX2*), a glutathione regulating enzyme (*GGT6*) and a serine protease that correlates with improved survival in ovarian cancer (*PRSS16*) [37]. The remaining 18 downregulated genes can be grouped into four functional categories: 1) Channel proteins (*ABCA13*, *ATP6VOA4*, *CACNA1F*, *GJB5*, and *TMC5*); 2) blood-antigen regulation (*ABO*, *B3GNT3*, *CD1A*, *FUT3*, and *ST6GALNAC1*); 3) GPCR/TKR signaling (*FAM83A*, *OR2C1*, *PAK6*, and *PLPP2*); and 4) cell‒cell/cell‒ECM interaction (*CORO2A*, *ITGB6*, and *MUC6*) (Table S11).

### 2.7. Quantitative PCR Supports the Accuracy of RNA Sequencing

We randomly selected five genes from those discussed above (*CCNB2*, *CYR61*, *CDC20*, *LIPE*, and *EPYC*) and performed quantitative RT-PCR to measure expression changes in four sets of matching pre- and post-NACT samples (Patients 1, 10, 16, and 17). In 85% of the comparisons (17 out of 20) the qRT-PCR results were in accordance with the results from RNA sequencing (Table 5).

## 3. Discussion

Our data demonstrate that there are multiple changes in gene expression profiles following exposure of HGSOC to platinum and taxane therapy and that these appear to develop early in the treatment of cancer. These changes are sufficiently similar between patients to distinguish pre- and post-treatment specimens, suggesting that biological pressure may be more relevant to the initial response to chemotherapy than the expansion of minor resistant subclones. A recent study by Arend et al. measured pre- and post-NACT expression of 770 cancer genes using the Nanostring platform [38]. Unsupervised clustering using the 86 differentially expressed genes from this panel of 770 genes resulted in a similar separation between pre- and post-NACT samples. Interestingly, when using all 770 genes, the samples did not cluster based on pre- and post-NACT status in the Arend study, while in our study, clustering using an expanded set of 6748 genes did result in separation (Figure 2). This highlights the differences between using an unbiased whole transcriptome approach versus a targeted panel analysis. There was strong concordance between the two studies, with 19 of the top 20 differentially expressed genes identified in the Arend study also significantly changed in our study.

A caveat to gene expression analysis using bulk tissue samples is the contribution of non-cancer cell types within the tumor microenvironment. Gene transcripts from infiltrating stromal, vascular and immune cells are inevitably mixed in with the cancer cell mRNA, producing a “bulk” gene expression signature. Several groups have demonstrated that small numbers of infiltrating cells, comprising as little as 5% of the total sample, will affect the gene expression signature [39,40], which can heavily influence the molecular subtype assigned to the patient [41]. Our samples had a similar range of tumor purity based on pathologic analysis and the ESTIMATEScore (Table 1, Figure S1) compared to TCGA samples [28]. It is highly likely that these infiltrating cells contributed to the gene expression signatures. Future studies using microdissected tissue and/or single cell analyses will be required to determine the effect of these infiltrating cells on the molecular signatures.

We were most interested in the pathways or biological states that were enriched in the post-NACT samples, as these could represent potential therapeutic windows if targeting those pathways could block the ovarian cancer cells’ ability to resist chemotherapy treatment. Interestingly, only three hallmark gene sets were enriched in the post-NACT samples compared to the pre-NACT samples: Bile acid metabolism, Adipogenesis, and Ultraviolet-response-down (Figure 5). Tumor purity may have affected these results, although changes in tumor purity comparing pre- to post-NACT samples were varied, with half of the samples showing increased purity and half showing decreased purity (Table 1, ESTIMATEScore change). However, three of the four samples from the omentum had decreased purity, which could indicate increased infiltration of adipocytes, which could account for the enriched adipogenesis gene set.

The hallmark gene set with the strongest normalized enrichment score was the bile acid metabolism gene set. This gene set is based on 28 founder gene sets, including several datasets relating to transporters, peroxisome, and response to drug. To better understand this enrichment, we identified the specific genes that were upregulated in our samples that caused gene set enrichment. Of the 38 genes that contributed to this enrichment, the top 15 were characterized by drug transport, fatty acid metabolism, and peroxisome production genes (Table 6 and Figure 5B). Both the Adipogenesis hallmark enrichment and the fatty acid metabolism enrichment could be the result of changes in the cancer epithelial cells or, alternatively, infiltration of fat cells within the sample taken during interval debulking surgery. Five of the 15 genes contributing to the Bile Acid Metabolism hallmark are drug transporters. Although we did not identify upregulation of *ABCB1* in our analysis, there is evidence that upregulation of *ABCB1* occurs via fusion with upstream promoters [8]. It is possible that we did not detect upregulation of *ABCB1* because our sequence analysis pipeline rejected these transcripts due to non-alignment with annotated transcripts, or the upregulation did not change during the first three cycles of chemotherapy administration. Our data indicate that, in addition to *ABCB1*, other transporters, especially those in the ABC1 family of transporters, may be playing an important role in the ovarian cancer response to chemotherapy treatment. Finally, our data indicate that cancer cells respond to chemotherapy by increasing peroxisome activity. Peroxisomes are key hubs within the cell for controlling reactive oxygen species [42,43]. Together these results suggest that inhibitors of drug transport, not just limited to ABCB1/MDR, and inhibitors of peroxisomes and fatty acid metabolism may be effective in blocking cancer cells’ response to chemotherapy.

Numerous studies have demonstrated a large degree of heterogeneity in gene expression patterns found in HGSOC patients [8,20], which presumably would result in pre-NACT and post-NACT samples from the same patient clustering together due to their unique pattern of copy number changes. In contrast to this prediction, unsupervised hierarchical clustering and k-means clustering, using the most variably expressed genes, indicated that the effects of chemotherapy on gene expression appear to outweigh the effects of inter-patient variability (Figure 2). Arend et al. performed a similar study using a targeted gene panel and also found that pre- and post-NACT samples cluster together [38]. A previous study comparing the change in chromosomal alterations between matched pre-NACT and post-NACT samples found that there were no significant changes in the chromosomal architecture during the first three cycles of chemotherapy [44]. These findings combined with our findings of extensive gene expression changes indicate that chemotherapy has a stronger effect on gene expression than clonal evolution within the short time frame of NACT. 

Currently there are no clinically useful biomarkers or gene expression patterns that predict response to carboplatin and paclitaxel [7,45]. Analyses of gene expression in large cohorts of HGSOC patients has led to classification of patients into molecular subtypes based on various unsupervised clustering algorithms. Several groups have proposed between four and nine molecular subtypes for ovarian cancer based on gene expression and other omics datasets in hope of improving patient outcomes through tumor-directed therapy selection [8,19,20,21,23,24,25]. While appealing, especially for the treatment of patients who are categorized as likely to be platinum-resistant, one problem with this approach is that the patient cohort and the platform for measuring gene expression can affect the robustness and applicability to other datasets. Our group has previously demonstrated that the technology used to measure gene expression can affect placement of patients into their molecular subtype. When we re-analyzed the original TCGA cohort using RNASeq gene expression data, instead of the original microarray gene expression data, a large percentage of the patients were classified into different molecular subtypes [46]. Due to this and other factors, it is difficult to make robust molecular classifications based on gene expression [47]. In this present study, we demonstrate that classification of patients into specific molecular subtypes changes after administration of chemotherapy (Figure 4 and Table 2). One caveat to this analysis is that our samples were from metastatic sites (Table 1) and not from the primary ovarian tumor. The TCGA molecular subtypes were based on gene expression in the primary ovarian tumor and it has not been established that gene expression from metastatic sites or from post-NACT samples can be applied to the molecular subtype stratification. Nevertheless, our data suggest that the subtypes may not be as useful as has been proposed because a patient’s classification into a subtype might change after exposure to chemotherapy. Furthermore, the change is not predictable based on the initial classification, as the three patients classified in the Immunoreactive subtype were all assigned to different subtypes after NACT.

Unsurprisingly, pre-NACT samples were notably enriched for multiple pathways involving cell cycle progression (Table 3, Tables S3 and S4) consistent with the concept that HGSOC is a highly proliferative disease and that platinum and taxane therapy reduces proliferation dramatically. This is further supported by the finding that common proliferation markers, *MKI67* and *PCNA* were both significantly downregulated in the post-NACT samples (Tables S2 and S5). A subset of ovarian cancer is known to overexpress *CCNE1* or *CCND1* [8], suggesting that cell-cycle-targeted drugs such as palbociclib, a CDK4/6 inhibitor, may be effective in specific subsets of ovarian cancer [48]. In our study, the levels of *CCND1*, *CCND2*, *CCNE1* and *CCNE2* were relatively unchanged after treating with chemotherapy, while *CCNB* and *CCNA* were significantly downregulated (Table S12), suggesting cell cycle inhibitors may be effective even in patients without elevated *CCNE* or *CCND*.

## 4. Materials and Methods 

### 4.1. Patient Recruitment 

After obtaining study approval by the Institutional Review Board (IRB number 1402M48375, approved 5/6/2014), we recruited women over the age of 18 with clinical, laboratory and/or imaging findings suspicious for advanced (FIGO Stage IIIC or IV) epithelial ovarian cancer treated at the University of Minnesota Medical Center. Samples were collected at the time of confirmatory biopsy in the setting of planned neoadjuvant therapy or when optimal debulking was deemed unfeasible intraoperatively. Study enrollment occurred after histologic assessment demonstrated high grade serous ovarian cancer and NACT was finalized as the treatment plan. Patients with a non-epithelial ovarian cancer, borderline ovarian cancer, or unclear histology were excluded from the study based on pathologic diagnosis. Demographic and clinical factors, including age at diagnosis, comorbid medical conditions, disease stage, and tumor histology, as well as details regarding treatment and survival outcomes were abstracted from the medical record. All patients gave written informed consent prior to enrollment.

### 4.2. Sample Collection, RNA Extraction, Library Preparation, and Sequencing

Samples of tumor tissue were obtained in the operating room or the interventional radiology suite and then sent to pathology where the samples were divided for subsequent analysis. A portion of each tissue sample was immediately placed into 2.5 mL of RNAlater solution prior to subsequent RNA-Seq analysis. RNA was extracted using the RNeasy Micro Kit (Qiagen, Redwood City, CA, USA) following the manufacturer’s protocol. RNA was quantified using RiboGreen (Thermo Fisher Scientific, Waltham, MA, USA). RNA integrity was assessed using capillary electrophoresis via the Agilent BioAnalyzer 2100 (Santa Clara, CA, USA), generating an RNA Integrity Number (RIN). To proceed to sequencing, samples had to be at least 1 microgram and have a RIN of 8 or greater. RNA samples were converted to Illumina sequencing libraries using Illumina’s Truseq RNA Sample Preparation Kit (Illumina, San Diego, CA, USA) following manufacturer’s protocol. Briefly, 1 microgram of total RNA was purified using oligo-dT coated magnetic beads, fragmented and then reverse transcribed into cDNA. The cDNA was blunt-ended and ligated to indexed adaptors and amplified using 15 cycles of PCR. Indexed libraries were paired-end sequenced using an Illumina HiSeq 2500 instrument. Sequences were processed using the CASAVA workflow to produce Fastq files.

### 4.3. Pathology

Patient samples were fixed and paraffin-embedded following standard protocols. Slides prepared by hematoxylin and eosin staining were analyzed for tumor purity and Chemotherapy Response Score [26,27] by a board-certified pathologist with extensive experience in gynecologic malignancies (KM).

### 4.4. Analysis of RNA-Seq Data

Mapping and transcript abundance: RNA Seq data were aligned to the GRCh38 reference genome using Tophat with default options [49]. Reads were filtered based on a mapping quality score ≥30 and we required that sequences uniquely mapped to the genome. Transcripts were quantified using Cufflinks to calculate fragments per kilobase of exon per million fragments (FPKM) values and featureCount to calculate absolute read counts [49,50]. FPKM values for all genes and all samples are provided in Table S5.

Differential gene expression was determined using the EdgeR software package [33]. We used pairwise analysis for pre- versus post-NACT comparisons and selected genes with an FDR *p*-value <0.001 and fold change > +/− 1.5. We used an FDR *p*-value < 0.05 for sensitive versus resistant samples and the additional requirement that the minimum value − standard deviation in the upregulated group was greater than the maximum value + standard deviation in the comparison group. EdgeR uses an overdispersed Poisson model with empirical Bayes methods to account for both biological and technical variability when determining differentially expressed genes [33]. 

Hierarchical clustering was performed using Cluster 3.0 and visualized using TreeView v. 1.1.6r4 using average linkage with a Euclidean similarity metric [51,52]. K-means clustering was performed using Cluster 3.0 with 1000 runs using a Euclidian Distance similarity matrix. Genes used for clustering were selected based on expression levels > 5 fpkm and an average deviation > 50 when comparing pre- to post-NACT samples (366 genes fulfilled these criteria). Average deviation was calculated using the Avedev function in Microsoft Excel, which calculates the average of absolute deviations from the mean in a given set of data. Principal component analysis was performed using the prcomp function in R, using genes that were consistently expressed in either all of the pre-NACT samples or all post-NACT samples, i.e., all samples had an FPKM > 5 (6748 genes fulfilled this criteria). Gene set enrichment analysis (GSEA) was performed using the MSigDB Hallmarks 50 gene sets (C1 v6.1), the Curated 4738 gene sets (C2 v6.1), and the Oncogenic Signatures 168 gene sets (C6 v6.1) [31,32]. GSEA uses a combination of statistical tests to identify enrichment in these pre-defined hallmark gene lists. Overrepresentation Analysis was performed using the online tool, Consensus Site Pathway Database (CPDB) http://consensuspathdb.org [53]. The stromal, immune, and ESTIMATEScores were calculated using the ‘estimate’ R package v1.0.13.

### 4.5. Quantitative RT-PCR

Half a microgram of total RNA was reverse-transcribed using the SuperScript^®^ III First-Strand Synthesis SuperMix for qRT-PCR reverse transcription kit (Invitrogen Life Technologies, Carlsbad, CA, USA) following the manufacturer’s specifications. RT-qPCR was performed in triplicate using 10-fold diluted cDNA, FastStart Essential DNA Green Master mix (Roche, Basel, Switzerland), and specific primers for *CCNB2*, *CDC20*, *CCN1*, *LIPE* and *EPYC*. Samples were run in the LightCycler 96 (Roche). Data were normalized to human TATA-box binding protein and fold change was calculated using the delta‒delta Ct method. Primer sequences are included in Table S13. 

### 4.6. Statistical Considerations

This was a pilot project and the sample size was limited by budgetary constraints; as such, a sample size calculation was not undertaken and the demographic parameters are descriptive. 

## 5. Conclusions

Numerous changes in tumor gene expression profiles after exposure to NACT were identified in this pilot study. One provocative finding was that the response to chemotherapy was similar across all patients, suggesting a common evolution during chemotherapy. A second provocative finding was that the molecular subtypes changed, but not in a consistent direction, suggesting a plasticity that could hinder the ability to use these subtypes as prognostic or predictive tools. A caveat to this is that in our study molecular subtypes were inferred from metastatic samples, while the original subtypes were defined using primary samples. The study is also limited by its small sample size; therefore, no conclusions can be drawn regarding whether these changes are correlated with platinum resistance or whether they affect prognosis. Nevertheless, these intriguing findings raise many questions and warrant a closer and more detailed analysis of the immediate response to chemotherapy in future studies.

## Figures and Tables

**Table 1:** Patient characteristics.

Patient ID	Age at Diagnosis	Number of NACT Cycles	PFS ^1^ (Months)	Platinum Classification ^2^	Pre- and Post- Sample Site	CRS ^3^	ESTIMATE Score Change ^4^
1	47	3	5	Resistant	Omentum	2	+2309
9	56	7 ^5^	12	Sensitive	Omentum	3	−108
10	57	3	3	Resistant	Peritoneum	2	−3383
16	74	3	2	Resistant	Omentum	1	+1898
17	58	3	10	Sensitive	Omentum	3	+655
22	78	3	5	Resistant	Peritoneum	2	−2070

^1^ PFS, progression-free survival. Patients 9 and 17 had not suffered a relapse at time of publication. ^2^ Platinum classification: Resistant = disease progression within six months of final chemotherapy administration. Sensitive = no disease progression within six months of final chemotherapy administration. [1]. ^3^ CRS, Chemotherapy response score: 1 = no or minimal tumor response, 2 = appreciable tumor response, 3 = complete or near-complete response. Based on Bohm et al., 2015 [26]. ^4^ Absolute change in ESTIMATEScore comparing post- to pre-NACT samples [28]. ^5^ Patient was responsive to platinum-based therapy but was medically unfit for surgery until the completion of seven cycles of chemotherapy.

**Table 2:** Molecular subtype assignment.

Patient ID	Pre-NACT Molecular Subtype	Post-NACT Molecular Subtype
1	Proliferative	Mesenchymal
9	Mesenchymal	Differentiated
10	Immunoreactive	Proliferative
16	Immunoreactive	Immunoreactive
17	Differentiated	Differentiated
22	Immunoreactive	Mesenchymal

NACT, neoadjuvant chemotherapy. Red ID indicates platinum-resistant patients, green ID indicates platinum-sensitive patients.

**Table 3:** Hallmark gene sets enriched in pre-NACT samples.

Hallmark Name	Size	ES	NES	NOM *p*-val *	FDR *q*-val *
G2M CHECKPOINT	151	0.76	1.89	0.00	0.003
E2F TARGETS	166	0.78	1.82	0.00	0.004
MTORC1 SIGNALING	158	0.60	1.68	0.01	0.028
GLYCOLYSIS	162	0.59	1.70	0.01	0.029
UNFOLDED PROTEIN RESPONSE	92	0.53	1.54	0.04	0.079
DNA REPAIR	114	0.50	1.55	0.01	0.085
MYC TARGETS V1	159	0.60	1.55	0.05	0.100
MITOTIC SPINDLE	163	0.49	1.47	0.04	0.112
MYC TARGETS V2	51	0.62	1.47	0.11	0.125
PI3K AKT MTOR SIGNALING	78	0.48	1.42	0.05	0.153

* *p*-val is calculated by comparing the observed ES score with a set of ESnull scores computed with randomly assigned phenotypes. The FDR *q*-val is based on a null distribution of NES scores [31].

**Table 4:** Hallmark gene sets enriched in post-NACT samples.

Hallmark Name	SIZE	ES	NES	NOM *p*-val	FDR *q*-val
BILE ACID METABOLISM	89	0.53	1.76	0.002	0.007
ADIPOGENESIS	155	0.46	1.65	0.000	0.012
UV RESPONSE DN	110	0.44	1.49	0.002	0.049

**Table 5:** Comparison of qRT-PCR results with RNA sequencing results.

Gene Symbol	Patient ID	Fold Change Based on qRT-PCR	Fold Change Based on FPKM	Change in Same Direction
CYR61	1	7.3	14.1	yes
CYR61	10	−2.2	−1.9	yes
CYR61	16	−7.1	20.7	no
CYR61	17	20.3	3.5	yes
CCNB2	1	−88.6	−48.9	yes
CCNB2	10	−5.6	−4.5	yes
CCNB2	16	−1.8	−7.9	yes
CCNB2	17	−4.1	−5.4	yes
CDC20	1	−9.0	−72.2	yes
CDC20	10	−2.5	−4.4	yes
CDC20	16	0.7	−14.8	no
CDC20	17	−10.7	−7.8	yes
EPYC	1	−44.9	−44.6	yes
EPYC	10	−4.2	−10.6	yes
EPYC	16	5.9	2.6	yes
EPYC	17	−340.1	−166.5	yes
LIPE	1	186.1	168.6	yes
LIPE	10	12.6	2.4	yes
LIPE	16	2.3	31.0	yes
LIPE	17	0.4	−2.6	no

**Table 6:** Top 15 upregulated genes in post-NACT samples contributing to enrichment in the Hallmark Bile Acid Metabolism gene set.

Gene Symbol	Function
CAT	Antioxidant, Catalase enzyme
CH25H	Cholesterol metabolism
RETSAT	Drug metabolism
ABCA5	Drug transport (ABC1 family)
ABCA6	Drug transport (ABC1 family)
ABCA8	Drug transport (ABC1 family)
ABCA9	Drug transport (ABC1 family)
ABCD2	Drug transport (ALD family)
ISOC1	Enzymatic production of pyruvate
LIPE	Fatty acid and cholesterol metabolism
HACL1	Fatty acid metabolism
PECR	Fatty acid metabolism
PEX11A	Peroxisome membrane elongation
BMP6	Regulates bone development and ovulation, secreted TGFb ligand
AR	Steroid hormone receptor, transcription factor

## Supplementary Materials

Supplementary materials are available online at https://www.mdpi.com/1422-0067/20/9/2131/s1.
